# Understanding the determinants of vaccine hesitancy in the United States: A comparison of social surveys and social media

**DOI:** 10.1371/journal.pone.0301488

**Published:** 2024-06-06

**Authors:** Kuleen Sasse, Ron Mahabir, Olga Gkountouna, Andrew Crooks, Arie Croitoru

**Affiliations:** 1 Department of Computer Science, The Johns Hopkins University, Baltimore, Maryland, United States of America; 2 Geographic Data Science Lab, Department of Geography and Planning, University of Liverpool, Liverpool, United Kingdom; 3 Department of Geography, University at Buffalo, Buffalo, New York, United States of America; 4 Department of Computational and Data Sciences, George Mason University, Fairfax, Virginia, United States of America; University of Hong Kong, HONG KONG

## Abstract

The COVID-19 pandemic prompted governments worldwide to implement a range of containment measures, including mass gathering restrictions, social distancing, and school closures. Despite these efforts, vaccines continue to be the safest and most effective means of combating such viruses. Yet, vaccine hesitancy persists, posing a significant public health concern, particularly with the emergence of new COVID-19 variants. To effectively address this issue, timely data is crucial for understanding the various factors contributing to vaccine hesitancy. While previous research has largely relied on traditional surveys for this information, recent sources of data, such as social media, have gained attention. However, the potential of social media data as a reliable proxy for information on population hesitancy, especially when compared with survey data, remains underexplored. This paper aims to bridge this gap. Our approach uses social, demographic, and economic data to predict vaccine hesitancy levels in the ten most populous US metropolitan areas. We employ machine learning algorithms to compare a set of baseline models that contain only these variables with models that incorporate survey data and social media data separately. Our results show that XGBoost algorithm consistently outperforms Random Forest and Linear Regression, with marginal differences between Random Forest and XGBoost. This was especially the case with models that incorporate survey or social media data, thus highlighting the promise of the latter data as a complementary information source. Results also reveal variations in influential variables across the five hesitancy classes, such as age, ethnicity, occupation, and political inclination. Further, the application of models to different MSAs yields mixed results, emphasizing the uniqueness of communities and the need for complementary data approaches. In summary, this study underscores social media data’s potential for understanding vaccine hesitancy, emphasizes the importance of tailoring interventions to specific communities, and suggests the value of combining different data sources.

## Introduction

The impact of the COVID-19 pandemic has resulted in governments worldwide having to implement a slew of different measures to contain the spread of the disease. Such measures, for example, included canceling mass gathering activities, mandating social distancing, school closures, and travel restrictions [1]. However, while these efforts have had some efficacy in slowing disease spread, vaccinations still remain the safest, most effective, and viable approach [2, 3]. As of January 2024, more than 13.5 billion doses of a COVID-19 vaccine had been administered globally. This has resulted in about 71% of the world population having received at least one dose of a vaccine, and 65% being fully vaccinated [4]. Previously, adults 80 years or older were found to be more predisposed to COVID-19; following which, there was increased susceptibility in young adults (aged 18–24 years), and among children and adolescents (aged 0–17 years) [5]. Now, several variants later, with new variants on the rise [6] and with recent COVID-19 outbreaks reported [7], it’s critical more than ever to vaccinate in order to continue slowing the disease’s transmission. This is vital so that those within the population who cannot be vaccinated, including the very young and immunocompromised, are still protected [8]. Further, while achieving herd immunity may not be feasible due to the evolving nature of the virus [9, 10], ongoing vaccination efforts remain essential to mitigating its impact and safeguarding public health.

Globally, while the total number of people that have received a COVID-19 vaccine has improved over time, these numbers vary by country. In the US, for example, only about 67% of the US population have been fully vaccinated [11]. However, at the state level, these numbers vary, with the lowest and highest vaccination rates being 52.8% (i.e., Wyoming) and 92.2% (i.e., District of Columbia) respectfully [12], and with even further discrepancy at more disaggregated spatial levels. Such variations in vaccination rates within and amongst countries have prompted investigations of the underlying factors that lead people to delay acceptance, or refuse vaccines despite their availability, a phenomenon referred to as vaccine hesitancy [13]. Factors that have been associated with vaccine hesitancy include ethnicity, working status, religious beliefs, political views, gender, age, education, income [14], online misinformation [15], and specific moral values [16]. Yet the majority of such work has depended on conventional surveys to collect data from individuals or groups using questionnaires or interviews that can be in-person (e.g., [17]), online (e.g., [18]), or over the phone (e.g., [19]). While these data sources have contributed to a large and diverse knowledge base on vaccine hesitancy, it’s important to consider their limitations (e.g., various biases or sample size) which may limit the applicability and effectiveness of the results (discussed further in the next section).

More recently, newer sources of data, in particular, social media, has emerged as a promising source of information on vaccine hesitancy. Currently, about 60% of the global population (i.e., 4.9 billion people) use social media services, such as X (formerly known as Twitter and henceforth used interchangeably) and Facebook, with this number expected to increase to 5.9 billion people by 2027 [20]. During the COVID pandemic, many people turned to social media as a way of keeping connected and informed about the pandemic [21]. Twitter, in particular, saw a 10.3% increase in users from 2019 to 2020 (the peak of pandemic) [22]. Other social media platforms, such as Facebook, also saw a notable increase of 8.7% [23]. The large number of users on social media makes it a rich source of information on peoples’ opinion and sentiments towards various topics, including the pandemic, providing valuable insights into public attitudes and trends [24, 25].

Although there there exists a substantial body of research that have utilized social media data to examine various aspects of vaccines, much of the research have focused only on a few themes. These include analyzing misinformation campaigns and particular communities, such as the anti-vaccination movement (e.g., [26, 27]), exploring the network interactions among hesitant community members (e.g., [28, 29]), understanding sentiments towards vaccines (e.g., [30, 31]) and the role of social media in influencing public attitude towards them (e.g., [32–34]), and analyzing topics of discourse surrounding vaccines (e.g., [35, 36]). However, the potential of social media data as a viable proxy for understanding vaccine hesitancy and its underlying determinants, particularly when juxtaposed against traditional survey data, remains an area that has garnered relatively little attention. Such an investigation is of significant importance given the profound and ongoing impacts of COVID-19 on our society, and the increasing influence of social media in our interconnected digital age. Further, given that pandemics are expected to continue to occur [37], it is important to continue to identify opportunities for collecting reliable information at scale, reasonable cost, and in a timely manner to help inform public health strategies and interventions. To address this gap, the primary research objective of this study is to evaluate the utility of social media data as a proxy measure for understanding vaccine hesitancy. Additionally, we aim to explore the determinants of vaccine hesitancy using modern machine learning approaches across a broader geographical scope compared to previous work.

## Related work

Although research on vaccine hesitancy is not new [38, 39], the advent of the recent COVID-19 pandemic has sparked a massive resurgence of interest surrounding this topic. In particular there has been a growing interest in understanding the specific reasons behind individuals’ reluctance to accept the COVID-19 vaccine. Work by [14], for example, delved into the pertinent literature and identified several key factors contributing to vaccine hesitancy. These include apprehension arising from the expedited development of vaccines, the perception of minimal risk regarding the disease due to prior immunization, skepticism surrounding the origin and efficacy of existing vaccines, and a pervasive lack of confidence in the institutions responsible for their production and distribution. Similar findings have been reported by [18, 40–42] with respect to individual determinants of vaccine hesitancy. Other research have found that a lack of time to get a vaccine [43], distrust in the political entities advocating for vaccinations [44], infringements on individual autonomy regarding vaccine accessibility at some locations, conspiracy theories [45], and commercial profiteering [46], further result in increase hesitancy rates. Studies have additionally reported hesitant populations within more specialized groups such as medical professionals (e.g., doctors and nurses) [47], and parents that have not cared for positive COVID-19 cases [48]. Moreover, work by [49, 50] found that even when provided with scientific information to support the efficacy and safety of vaccines, some parents still opt to not vaccinate their children. This suggests that much broader social and cognitive processes may be at play when it comes to making a decision on whether or not to vaccinate [51].

Most of the aforementioned studies have relied on the use of traditional survey instruments, through in–person meetings or online surveys, to elicit insights into vaccine hesitancy. However, these approaches come with inherent limitations that pose challenges in effectively gathering data about vaccines and understanding individuals’ reservations towards them. As it relates to in–person surveys; these are very labor intensive, time consuming, and require substantial financial resources [52]. Such costs can be as much as $40 per-person for in-person surveys, or $22 per person for more cost effective options, such as the use of mobile phones using interactive voice response [53], and with data collection costs increasing [54]. This makes it difficult, or at least very costly, to up-scale such work to large geographical scales.

Another concern that has been recognized is that of biases. In the acquiescence bias, survey respondents exhibit a tendency to favor positive response options or express a positive sentiment in a disproportionately frequent manner [55]. Previous work by [56] using China and the US as case studies, for example, showed that acquiescence bias can inflate estimated incidence of conspiratorial beliefs and political perceptions by as much as 50%. Related to this is the dissent bias where people tend to express a negative agreement in a more frequent manner [57]. Survey results can also be affected by social desirability bias wherein respondents choose responses that they believe will make them be viewed favorably by others [57].

In addition to these concerns, traditional surveys face a range of challenges related to participation and timeliness. Declining response rates over time [58], can lead to issues with unit non-response, where participants do not respond to all questions or do not provide enough information for the response to be deemed usable. This is further compounded by the issue of item non-response, in which participants respond to questions but do not provide a usable response to a particular item or items [59]. Beyond participation, surveys can suffer from problems of temporal relevancy, which represents the need to have data collected as close as possible in time to the event of interest [60, 61]. As such, they must be planned ahead of time, which would typically mean having knowledge about an event that is either yet to happen, or the ability to collect participants’ information on short notice following an event. For mass emergency and rapidly unfolding events, such as natural disasters and epidemics, advanced prior knowledge may be limited, and participants may otherwise be pre-occupied during these times to take part in surveys. Further, because surveys typically represent the current view of participants as of the date of the survey, they are unable to adequately address issues from bias stemming from experiences that may have occurred prior to the administered survey date [60]. For example, data collected from people after they have had severe side effects from a vaccine may lead to a negative view towards vaccines, and could influence other members within their social circle to not vaccinate. Collectively, these biases can convolute public opinion, potentially leading to distorted interpretations of the true beliefs and perceptions landscape.

In light of the shortcomings of traditional surveys, other sources of data have been explored. One such source is social media data collected from online platforms such as Twitter and Facebook. Compared to survey data, social media presents several advantages, including the ability to scale data collection efforts at reasonable cost, permit archival searches to capture more temporal relevant data surrounding events of interest, as well as other relevant information that were not included in the original survey instrument [60]. With respect to the latter, whereas surveys tend to be restricted to what is required, thus leading to a higher possibility of issues with unit and item non-response, the content on social media remains largely unbounded. This, in turn, can increase the potential for capturing additional useful information about the particular event or phenomenon that can be of value. These benefits are also expected to extend to instances of acquiescence and dissent bias as well, with social media allowing for more freedom of expression [62], compared to the use of poorly constructed survey instruments that typically lead to such issues [63].

Prior research on surveys has demonstrated that respondents experience lower social anxiety and social desirability when participating in online surveys as opposed to face–to–face interactions, which can be attributed to the heightened anonymity provided by the virtual environment [64]. In the case of social media platforms, such as Twitter, individuals have the option to adopt pseudonyms, helping to safeguard their real identity [65] and thereby helping to reduce these concerns. Further, during the pandemic, many people turned to social media as a way of combating depression and anxiety manifested from the event; discussing a range of different topics, including vaccines, along with individuals’ perspectives, beliefs, and attitudes toward them [45, 66]. This makes social media a rich source of information on vaccine hesitancy that can be curated and analysed, providing important insights that can be used to better understand this issue. Finally, from a more technical perspective, many social media platforms provide a dedicated Application Programming Interface (API), offering a flexible pathway for data retrieval that is often not available in conventional survey methodologies [67].

When it comes to vaccine hesitancy, several studies have explored this subject through the lens of social media. [68], for example, applied topic modelling to Twitter messages to gather information on the contributing factors of immunization uptake. That study identified various factors relating to access (e.g., location of vaccine), affordability (e.g., price of additional services), awareness (e.g., knowledge about vaccines), acceptance (e.g., perceived vaccine safety), activation (e.g., incentives), and assurance (e.g., protection) for variations in uptake. Similar applications of topic modelling include work by [35, 36, 69, 70]. A number of studies have also applied sentiment analysis to social media to gauge the general sentiment and attitudes of individuals towards vaccines and vaccination efforts [30, 71–74]. Work by [30, 31], for instance, used sentiment analysis to study the public’s emotional stance surrounding the pandemic; with the public mainly having a negative view. More recently, research by [75] explored the association between vaccine hesitancy rates and socio-demographic characteristics derived from survey and social media data (i.e., Twitter). This study achieved notable accuracy levels for age (91%), gender (75%), and political ideology (77%). Moreover, a comparison of vaccine hesitancy figures from both survey data and Twitter posts across different time frames yielded Pearson’s correlation coefficients in the range of 0.57 to 0.8.

Additional work by [26, 27] have studied misinformation campaigns about the pandemic. Those studies show that anti–vaccination communities on Twitter leaned mainly to the far right direction of the political spectrum, with references to websites and content with already questionable credibility. Moreover, [28, 29] studied the network interactions of members in vaccine–hesitant communities to understand their scale of impact, and the specific topics that were being propagated on social networks. Such studies, while informative, have mainly used the textual content embedded within social media posts to understand contributing factors towards hesitancy, missing the equally important social, demographic, and economic factors that also play a role in increased hesitancy rates [76].

In an attempt to address this issue, several studies have explored the use of such data for understanding the determinants of vaccine hesitancy. Studies by [77–79], for example, have identified low income, race, and level of education, to be important socio-demographic factors influencing hesitancy. Such work, however, have mainly relied on the use traditional surveys to collect this data; thus being exposed to some of their aforementioned issues with their use. Many countries collect large amounts of social, demographic, and economic data as part of national population census surveys, providing the ability to study such factors at scale. [80], for example, classified various socio-demographic variables into high and low socioeconomic groups to study topic prevalence within each group. That study showed that whereas the high group focused primarily on topics surrounding getting the vaccine, the low group mainly discussed an urgent need for medical and government support for the vaccine. Other work by [81] further built several regression models to understand the link between populations of unvaccinated persons and various socio–economic variables using census tracks data for the state of Texas in the US. That study reported main determinants to be neighborhoods with lower socio–economic standing and communities with signs of distrust in government.

Further work by [61] explored the performance of various machine learning algorithms using Twitter data for predicting vaccine hesitancy at the zip code level in the US. In that study, variables derived from Twitter messages (i.e., hashtags and sentiment score) were combined with different social, demographic, and economic variables (i.e., real estate value and number of different health, educational, professional, scientific and technical service providing establishments) and used to predict vaccine hesitancy derived from Gallup poll survey data. That study found that while there was an improvement in model performance with the inclusion of Twitter data, overall performance was low, with reported root mean square error values between 0.3 and 0.4. More recent work by [82] further analysed the spatial and temporal impacts of neighbourhood variables on COVID-19 outbreaks. The results of that work showed the proportion of Hispanic residents, residents with earnings below the poverty line, and residents ages fifteen to twenty–four to have high correlation with high incidence of disease. Moreover, [51] extracted topics from both Twitter and survey data to compare co–variation in belief in vaccine hesitancy. Using tweets to infer stance (i.e., level of agreement or disagreement), the authors concluded that there was good qualitative agreement between the first principal component loading and scores using survey and Twitter data.

Most studies combining these forms of social data and social media data, while providing a more holistic view of vaccine hesitancy, do not directly examine the value of social media, as compared to the use of survey data, when trying to understand this issue. Several studies have examined this question for different areas, including, health [83], the economy [84], and entertainment [85], but to the authors’ knowledge, there has been limited work with respect to vaccine hesitancy. The one exception is the study by [61]. However, as mentioned earlier, the reported accuracy values in that study were very low. Also, the data used were limited to real estate and types of establishments, providing an opportunity to explore other social, demographic, and economic variables, including those often investigated in related studies on vaccine hesitancy. Further, our study covers a much larger geographical area and integrates modern machine learning and deep learning approaches as part of our methodological workflow.

## Methodology

Our research methodology involves the collection and preprocessing of two primary data sources: public opinion survey data concerning vaccine hesitancy and relevant Twitter data. These data were gathered within the geographical scope of the US, focusing specifically on understanding the attitudes and sentiments towards COVID-19 vaccination among US participants. The survey data captured the inclination of individuals receiving a COVID-19 vaccine. On the other hand, Twitter data, obtained from various repositories, were categorized into three distinct hesitancy stances: “pro” (favoring vaccines), “anti” (opposing vaccines), or “neutral” (neither favoring nor opposing vaccines). These two data sources were collected to facilitate a comparison between baseline models for different hesitancy groups. These models utilized only socio-demographic and economic variables, while others were augmented with either survey data or social media data. All data were collected at the county level, which aligns with the analytical focus of our study. The mixed methods matrix presented in Table 1 provides an overview of the steps undertaken, illustrating the integration of socio-demographic and economic data with survey data, and with Twitter data to enhance our understanding of vaccine hesitancy. These steps (i.e., data collection, data processing, and model development and comparison) are discussed in greater detail in the subsections that follow.

**Table 1:** Mixed methods matrix showing the data, processing, and model development steps used in our study.

Data collection	Data processing	Model development and comparison	Outcome
COVID-19 vaccine hesitancy rates	(1) Extract rates for different hesitancy groups at the county level within each MSA study area.		(1) Hesitancy rates for different groups used to build our baseline models.
Socio-demographic and economic	(1) Review literature and identify factors related to vaccine hesitancy.	(1) Develop a set of baseline models, each representing a specific hesitancy rate group based on socio-demographic and economic variables.	(1) Baseline models used for explaining each hesitancy group.
	(2) Identify variables from relevant data sources.		
	(3) Extract variables at the county level within each MSA study area.		
	(4) Selection of most relevant variables for each hesitancy group.		
Survey	(1) Extract percentage of people within each hesitancy group at the county level within each MSA study area.	(1) Integrate survey data into each baseline model.	(1) Models highlighting the added value of incorporating survey data to each baseline model.
Social media	(1) Collect labelled tweets on vaccine hesitancy for different hesitancy stances.	(1) Use labelled tweets to build a multi-class model to classify tweets for each hesitancy stance.	(1) Models highlighting the added value of incorporating social media data to each baseline model.
	(2) Collect unlabelled tweets on vaccine hesitancy.	(2) Apply model to unlabelled tweets.	
	(3) Extract tweets at the county level within each MSA study area.	(3) Extract percentage of tweets within each hesitancy stance at the county level within each MSA study area.	
	(4) Integrate social media data into each baseline model.		

### Data

As previously discussed, studies have reported multiple reasons influencing an individual’s decision to not vaccinate. Therefore it was important to first identify these variables that contribute to vaccine hesitancy in order to develop our baseline models. To accomplish this, we first conduct an in-depth literature survey using online scholarly databases that included Google Scholar [86], Web of Science [87], and Scopus [88] to identify variables relevant to vaccine hesitancy. Following the compilation of variables, applicable data sources were identified for the US as shown in Table 2. The collection of data was undertaken at the county scale. In instances where county-level data was unavailable, data at a higher spatial scale (e.g., zip code) was gathered and subsequently aggregated to the county level using a summative approach. With the exception of the social vulnerability variable [89], all other were data provided as counts of people. The social vulnerability variable is an index that measures the level of concern for a difficult roll-out on a range from 0 (lowest concern) to 1 (highest concern). This data includes multiple characteristics of the people that live in counties.

**Table 2:** Data sources used in our study.

Variable	Description	Data source	Reference	Year(s)
Age	Number of persons in different age groups	American Community Survey, US Census Bureau	[90]	2019
Ethnicity	Number of persons in different ethnic groups	American Community Survey, US Census Bureau	[90]	2019
Education	Number of persons at different education levels	American Community Survey, US Census Bureau	[90]	2019
Cohabitation	Number of couples cohabiting	American Community Survey, US Census Bureau	[90]	2019
Occupation	Number of people in different job classifications	US Bureau of Labor Statistics	[91]	2021
Employment	Number of people employed and unemployed	US Bureau of Economic Analysis	[92]	2021
Income	Number of people in different income groups	American Community Survey, US Census Bureau	[90]	2019
Political party	Number of people that have voted for a specific political group	Harvard Dataverse	[93]	2020
COVID-19 cases	Number of COVID-19 cases	The New York Times	[94]	2021
COVID-19 vaccination	Percentage of people that have received the primary dose of a COVID-19 vaccine	Center for Disease Control	[95]	2021
COVID-19 vaccination	Percentage of people that have received the complete series of a COVID-19 vaccine	Center for Disease Control	[95]	2021
Social vulnerability	Social Vulnerability Index	Center for Disease Control	[96]	2021
Social vulnerability	Level of concern of vaccine rollout (value between 0 and 1)	Surgo Ventures	[89]	2021
Survey	Percentage of people that are vaccine hesitant	US Department of Health and Human Services	[97]	2021
Survey	Percentage of people that answered yes to specific questions on vaccines	Delphi survey, Carnegie Mellon University	[98]	2022
Social media	Twitter posts labelled as Pro-vaccine, Antivaccine, and Neutral	Twitter	[99]	2015 to 2021
Social media	Twitter posts labelled as Pro-vaccine, Antivaccine, and Neutral	Twitter	[100]	2015 to 2020
Social media	Twitter posts labelled as Pro-vaccine, Antivaccine, and Neutral	Twitter	[101]	2019 to 2021
Social media	Twitter posts labelled as Pro or Neutral	Twitter	[102]	2017 to 2020
Social media	Unlabelled Twitter posts	Twitter	[103]	2016 to 2021
Boundaries	Geographic boundaries—217 counties accross 10 metropolitan statistical areas	American Community Survey, US Census Bureau	[90]	2021

County level vaccine hesitancy rates were collected from the US Department of Health and Human Services (HHS) [97]. This data consisted of estimated COVID-19 hesitancy rates for each county in the US. To generate these estimates, the data initially utilized the Census Bureau’s Household Pulse Survey (HPS) data at the state level and subsequently extrapolated county-level rates using the Census Bureau’s 2019 ACS Public Use Microdata Sample (PUMS). HPS participants were asked if they would receive the COVID-19 vaccine when it became available. Five responses were captured: “definitely get a vaccine”, “probably get a vaccine”, “unsure”, “probably not get a vaccine”, and “definitely not get a vaccine”. Responses were used by HHS to compute data for three hesitancy groups: “strongly hesitant,” “hesitant,” and “hesitant or unsure.” The *strongly hesitant* group refer to people that stated they would “definitely not” receive a COVID-19 vaccine. The *hesitant* group refer to people that indicated that they would “probably not” or “definitely not” receive a COVID-19 vaccine. Finally, the *hesitant or unsure* group refer to people that stated they would “probably not” or “unsure” or “definitely not” receive a COVID-19 vaccine. Further, we use the HHS data to derive two additional group measures. The *unsure* group was derived by subtracting the percentage of people that answered that they would “probably not” or “definitely not” receive a COVID-19 vaccine from the *hesitant or unsure* group. The *probably not* group was derived by subtracting the percentage of people that stated they were “unsure” or “definitely not” receive a COVID-19 vaccine from the *hesitant or unsure* group. Computed values for these hesitancy groups represent our five target variables in this work.

Survey public opinion data were collected from the Delphi Survey, a product from the Delphi group at Carnegie Melon University [98]. The survey asked US participants, if a vaccine to prevent COVID-19 were offered to you today, would you choose to get vaccinated? Responses were: (1) Yes I would definitely choose to get vaccinated, (2) Yes I would probably choose to get vaccinated, (3) No I would probably not choose to get vaccinated, or (4) No I would definitely not choose to get vaccinated. This data were used by the Delphi group to compute two measures of hesitancy, and made available for public use. The “somewhat’ group is computed as the proportion of respondents who answered “no” or “I don’t know” to the second question divided by the total number of respondents who gave any answer to the first question. The “all” group is computed as the proportion is the number of respondents who answered “yes”, “no” or “I don’t know” to the second question divided by the total number of respondents who gave any answer to the first question. Percentages of the population in each group were provided for each US county. There represent our two survey variables.

Our work also leverages two types of Twitter data. Labeled tweets were collected from several sources [99–102], which each categorized them as one of three different hesitancy stances:“pro”, “anti”, or “neutral”. The count distribution of these tweets were 8,213, 2,322, 7,017 for “pro”, “anti”, and “neutral” stances respectfully. In some instances, only tweet IDs were provided from the online repositories, whereas the corresponding tweeted message was required. To collect these messages, the tweet ID was utilized to access the Twitter API, and the corresponding text message was saved.

Unlabelled Twitter data consisted of tweets that include not only vaccine hesitancy-related tweets related to COVID-19, but also discussions about other diseases such as measles, mumps, and rubella, which similarly sparked online conversations on social media during this time frame [99, 104, 105]. This diversity in hesitancy across multiple diseases is also reflected in the labelled data as well. The unlabelled data were collected from the Twitter platform using their API. A set of keywords that included “vaccine”, “vax”, “vaccine hesitancy”, “vaccine hesitant”, “anti-vax”, “anti-vaxx”, “antivax”, and “antivaxx”, were employed to query the API. These keywords were chosen based on previous research that delved into vaccine hesitancy through social media analysis. For instance, [15] utilized both Twitter and Facebook data and concluded that vaccine-hesitant individuals are more likely to post vaccine misinformation online compared to other groups. Similarly, [106] reported a significant presence of vaccine-hesitant groups across popular social media platforms, posting anti-vaccination messages. Only English tweets within the US were considered in this work. All personally identifiable information were removed from both labelled and unlabelled tweets and records were anonymized to protect the privacy of individuals. Additionally, to ensure data integrity and consistency, a rigorous data preprocessing pipeline was implemented, including steps such as removing duplicates, filtering out irrelevant tweets, and standardizing text formats. Moreover, procedures were carefully taken to ensure compliance with Twitter’s terms of service and use of data usage agreements.

Finally, we collect administrative boundary data for the ten of the most populous Metropolitan Statistical Areas (MSAs) in the US [90]. It is important to note that these MSAs serve as our primary study areas. They represent well-defined geographic regions with substantial economic and social significance, each with their own specific local behaviour and regional trend [107]. Further, recognizing that social media use tends to be concentrated in regions with larger populations and greater technological access, known as the digital divide [108–110], the use of MSA as our study areas helps to reduce such concerns. The MSAs are Atlanta-Sandy Springs-Alpharetta (GA), Chicago-Naperville-Elgin (IL-IN-WI), Dallas-Fort Worth-Arlington (TX), Washington-Arlington-Alexandria (DC-VA-MD-WV), Houston-The Woodlands-Sugar Land (TX), Los Angeles-Long Beach-Anaheim (CA), Miami-Fort Lauderdale-Pompano Beach (FL), New York-Newark-Jersey City (NY-NJ-PA), Philadelphia-Camden-Wilmington (PA-NJ-DE-MD), and Phoenix-Mesa-Chandler (AZ). These will henceforth be labelled as Atlanta (Atl), Chicago (Chi), Dallas (Dal), Washington DC (DC), Houston (Hou), Los Angeles (LA), Miami (Mia), New York (NYC), Philadelphia (Phl), and Phoenix (Phx), respectfully. Readers interested in more details on the specific collection and processing steps for the various data used in this research are referred to their specific reference in Table 2.

### Data processing

Following the extraction of socio-demograhic and economic variables (discussed in the previous section), with the exception of the the social vulnerability variable, all other variables were transformed from the absolute number of individuals within that variable group to a percentage value. This was done using total population data for counties provided by the American Community Survey [90]. Since no information is provided on the number of people or percentage of the population within each level of concern sub-category for social vulnerability, this data could not be transformed into a percentage value. Nonetheless, many studies have used this variable as a seminal measure to understand vaccine hesitancy (e.g., [111, 112]); thus, the decision was made to keep it as a variable in this work.

After variables were transformed, there was need to identify the most relevant subset of variables for each baseline hesitancy target group (i.e., strongly hesitant, hesitant, hesitant and unsure, unsure, and probably not). As emphasized by [113], this process is important for eliminating irrelevant, noisy, or unreliable variables, ultimately improving predictions and/or minimizing model complexity. Moreover, this approach mitigates the risk of over-fitting and enhances model runtime efficiency. For determining the optimal subset of explanatory variables for modeling, the BorutaSHAP [114] algorithm was used. This process yielded five distinct subsets of variables.

The BorutaSHAP algorithm combines the advantages of both the Boruta [115] and SHapley Additive exPlanations (SHAP) [116] algorithms to identify the most optimal subset of explanatory variables. The Boruta algorithm operates through iterative comparison of the importance of original variables against shadow variables, which are created by shuffling the original variables. Variables demonstrating significantly lower importance than their shadow counterparts are excluded from the variable set, while those performing notably better than the shadow variables are retained [115]. In the context of BorutaSHAP, the SHAP metric is utilized to ascertain variable relevance [117], often resulting in improved overall accuracy compared to Boruta [118]. Prior research has indicated that BorutaSHAP serves as a reliable feature selection technique [119–122], making it suitable for application within this study.

Concerning the Twitter data, all unlabelled data were spatially clipped to each respective MSA study area resulting in an approximate count of five million tweets. To ensure data quality, tweets further underwent a cleaning process involving the removal of URLs, emails, and usernames, the expansion of contracted words, and the replacement of emojis with their corresponding textual descriptions as suggested by previous related work [123–125].

The Delphi survey data consisted of two hesitancy groups: ‘somewhat’ and ‘all’. The percentage of each group per county was used to compute the corresponding percentage for the respective MSA. This aggregation involved utilizing the weighted sum of the population percentages within each MSA, and computation for each MSA as follows. Let $C_{i}\in C\;\forall i\in 1,\ldots ,n$ where C is the set of all counties in an MSA. The percentage of interest within the metropolitan statistical area, *H*(*M*), is given by:

$$\begin{array}{c} H\left(M\right)=\frac{\Sigma_{i=1}^{n}\left(H\left(C_{i}\right)\times P\left(C_{i}\right)\right)}{\Sigma_{i=1}^{n}P\left(C_{i}\right)}, \end{array}$$

where *H*(*M*) represents the percentage of the population within an MSA, *H*(*C*_*i*_) is the percentage of interest for the *i*^*th*^ county of the MSA, and *P*(*C*_*i*_) is the total number of people for the *i*^*th*^ county of the MSA. Similar to the Twitter data, the computed percentages of hesitant population at the MSA level were added as an attribute to each county within the respective MSA.

### Model development and comparison

Leveraging the Twitter labeled dataset with text, a classification model was constructed utilizing BERTweet [126], a pre-trained English tweet language model [127]. The model development followed an 80/20 training/testing split strategy. For model refinement and the identification of optimal hyperparameters, the Ray-Tune optimization framework, employing a population-based scheduler, was used in Python [128]. The resulting model achieved an F-score of 0.83, a metric value consistent with previous studies indicating a good model fit [38, 101, 129, 130]. The developed classification model was then applied to unlabeled data, classifying tweets as “pro”, “anti”, and “neutral” respectfully. Following this, the percentage of tweets per MSA, for each hesitancy group, was computed. These values were then added as an attribute to all counties within the respective MSA. This was done in order to account for distributional differences in tweets within MSAs, and to identify their broad regional hesitancy patterns. Moreover, aggregating the data to a higher geographic scale allows for a larger sample size, improving the statistical power of the analysis. This is crucial for making confident inferences and identifying meaningful correlations with other variables used in this study.

Next, a series of baseline models, that only contain the social, demographic, and economic explanatory variables were developed and compared with models that were augmented with either the survey or social media data. Three distinct modeling techniques were employed for this comparison: linear regression, random forest regression, and XGBoost regression. These methods were specifically chosen as they allow for the comparison of similar performance metrics, namely, *R*^2^ and mean absolute value. Additionally, these modeling techniques are widely utilized in similar contexts and are all available within various open-source Python packages (i.e., Scikit-Learn [131] for linear regression and random forest, and xgboost [132] for XGBoost), which were utilized in this study.

For each modeling method, a set of three models aimed at predicting hesitancy were built, one for each target variable (i.e., ‘strongly hesitant,’ ‘hesitant,’ ‘hesitant and unsure,’ ‘unsure,’ and ‘probably not’). To illustrate, for the ‘strongly hesitant’ group of models, there were three baseline models, each corresponding to the three modeling techniques employed. Additionally, there were three models that incorporate our survey variables and another three that incorporate our social media variables for the same target variable, again aligning with the three modeling techniques. To assess model performance, each model underwent 500 randomized 80/20 training/testing data splits, and their average adjusted *R*^2^ accuracy was recorded.

Similar to recent work by [61], a significance analysis was also carried out to determine the effectiveness of the best model. We used the Mann-Whitney U test statistic [133] and compared the baseline models against their social media and survey counterparts to determine whether the performance of these later models can be attributed to chance alone. Specifically we compute the *p*-value from this test statistic using the distribution of average *R*^2^ values. To address the potential for inflated significance due to multiple testing, we applied the Bonferroni correction on the *p*-value threshold of 0.05. This adjustment reduces the *p*-value threshold below the 0.05 threshold to account for multiple tests. We found even with the correction that that there was no change in the significance of our results before and after applying the Bonferroni correction in terms of comparing adjusted *R*^2^ value of the models.

Moreover, we assess the extent to which models can be applied generally. To achieve this, we follow a similar approach to [134], initially partitioning the data by MSA. Subsequently, we employed all three models (baseline, social media, and survey) for every technique and each hesitancy group within each MSA study area. This allowed for comprehensive comparisons across regions. The evaluation of each model’s effectiveness was based on their respective *R*^2^ values.

## Results

The primary objective of this study was to evaluate the utility of social media data as a proxy measure for understanding vaccine hesitancy. This objective is assessed in the first two subsections that follow, where we compare the performance of models utilizing social media data with those using survey data. Through this analysis, we aim to determine the effectiveness of social media data in capturing and predicting vaccine hesitancy trends, along with exploring the influence of geographic variation.

The secondary goal was to explore the determinants of vaccine hesitancy using modern machine learning approaches. This objective is assessed in the final subsection, where we examine the factors contributing to vaccine hesitancy identified by the developed models. By leveraging machine learning techniques, we aim to identify key determinants such as demographic variables and socioeconomic factors that influence individuals’ attitudes towards vaccination.

### Model performance and significance analysis

The information presented in Table 3 provides an overview of the performance metrics for various developed models and techniques. These metrics encompass average root mean squared error (RMSE), adjusted *R*^2^, and the percentage change in adjusted *R*^2^ with the addition of the social media and survey data into the baseline models. The RMSE values demonstrate strong model fit across all hesitancy models, particularly for the unsure, probably not, and strongly hesitant models. However, a closer examination of models’ adjusted *R*^2^ values reveal larger variability among these values. Specifically, the range of adjusted *R*^2^ values spans from 50% to 95%, highlighting differences both between models and across techniques. The linear approach has the largest performance range, with adjusted *R*^2^ values spanning from 50.4% to 89.5%. The adjusted *R*^2^ range for both random forest and XGBoost is comparable, lying between 75.8% and 94.1% for random forest, and 73.1% and 92.5% for XGBoost. Notably, XGBoost is the best performing method with an average of 87.2% and a standard deviation of 0.06. Nonetheless, these figures align closely with those of random forest, having an average of 86.9% and a standard deviation of 0.07. Furthermore, XGBoost consistently outperforms the other two methods across all models, while random forest also demonstrates superior performance compared to the linear method for all models.

**Table 3:** Model performance (Bolded values represent the best performing model for each approach).

Method	Model	RMSE	Adjusted R^2^	% Improvement in Adjusted R^2^
**Hesitant**				
Linear	Baseline	0.0216	0.7384	N/A
Linear	Social media	0.0163	0.8480	10.97
Linear	Survey	0.0135	**0.8952**	15.68
Random Forest	Baseline	0.0146	0.8771	N/A
Random Forest	Social media	0.0123	0.9132	3.62
Random Forest	Survey	0.0103	**0.9392**	6.21
XGBoost	Baseline	0.0162	0.8491	N/A
XGBoost	Social media	0.0125	0.9113	6.23
XGBoost	Survey	0.0115	**0.9249**	7.58
**Unsure**				
Linear	Baseline	0.0091	0.5595	N/A
Linear	Social media	0.0097	0.5042	-5.53
Linear	Survey	0.0090	**0.5673**	0.79
Random Forest	Baseline	0.0069	0.7582	N/A
Random Forest	Social media	0.0068	0.7580	-0.02
Random Forest	Survey	0.0068	**0.7595**	0.13
XGBoost	Baseline	0.0071	0.7311	N/A
XGBoost	Social media	0.0070	0.7393	0.82
XGBoost	Survey	0.0069	**0.7503**	1.92
**Hesitant or unsure**				
Linear	Baseline	0.0247	0.7616	N/A
Linear	Social media	0.0240	0.7704	0.88
Linear	Survey	0.0172	**0.8840**	12.23
Random Forest	Baseline	0.0184	0.8673	N/A
Random Forest	Social media	0.0168	0.8899	2.26
Random Forest	Survey	0.0146	**0.9145**	4.72
XGBoost	Baseline	0.0204	0.8393	N/A
XGBoost	Social media	0.0172	0.8835	4.42
XGBoost	Survey	0.0148	**0.9129**	7.35
**Probably not**				
Linear	Baseline	0.0090	0.6741	N/A
Linear	Social media	0.0075	0.7698	9.58
Linear	Survey	0.0062	**0.8412**	16.71
Random Forest	Baseline	0.0051	0.8942	N/A
Random Forest	Social media	0.0039	0.9391	4.49
Random Forest	Survey	0.0038	**0.9416**	4.74
XGBoost	Baseline	0.0061	0.8488	NA
XGBoost	Social media	0.0046	**0.9164**	6.75
XGBoost	Survey	0.0046	0.9147	6.59
**Strongly hesitant**				
Linear	Baseline	0.0141	0.7270	N/A
Linear	Social media	0.0120	0.8008	7.38
Linear	Survey	0.0095	**0.8734**	14.64
Random Forest	Baseline	0.0112	0.8308	N/A
Random Forest	Social media	0.0097	0.8744	4.36
Random Forest	Survey	0.0084	**0.9051**	7.43
XGBoost	Baseline	0.0121	0.8023	N/A
XGBoost	Social media	0.0102	0.8597	5.74
XGBoost	Survey	0.0091	**0.8864**	8.41

Turning to the utility of models utilizing social media data versus those leveraging survey data, Table 3 highlights that, except for the probably not model, models utilizing survey data consistently outperform both baseline models and those utilizing social media data across all approaches. For the probably not model, the performance disparity between the XGBoost model using social media data and its survey data counterpart is marginal at 0.16% higher for the former. Likewise, with the exception of the unsure model, models using social media data outperform all baseline models. Concerning the unsure model, linear and random forest baseline models exhibit better performance, achieving slight increases in *R*^2^ values of 5.53% and 0.02%, respectively, in comparison to the social media model. Notably, the performance values for the linear method remain relatively low, ranging from 55% to 57%. A comparison between the performance of social media models and those utilizing survey data underscores differences in the range of 0.16% to 12%. The most substantial differences are observed for the linear method, with disparities for random forest and XGBoost ranging from 0.15% to 3.1%. More generally, our findings show that with the additional of social media or survey data, model performance can be improved by as much as 17%, dependent on the specific model and technique being used.

Additionally, Table 4 shows the outcomes of the significance analysis. This table demonstrates that models developed for unsure, employing both social media and survey data, exhibit p-values well below the 0.05 significance threshold. Compared with the performance outcomes shown in Table 3, it is evident that the improvements in performance in these unsure models are minimal compared to other hesitancy models, ranging from 0.13% to 1.92%. Consequently, we accept the null hypothesis in this instance, concluding that these models’ results can be attributed to random chance. Conversely, all other models present significantly small p-values, indicating robustness and results that are not contingent on random chance. These findings parallel the performance metrics in Table 3, wherein the models with the weakest performance are predicated on linear methods. In the context of XGBoost, with the exception of the probably not hesitancy model, this method continues to outperform all others.

**Table 4:** Significance analysis.

Method	Model	P-value
**Hesitant**		
Linear	Social media	9.57 × 10^−108^
Linear	Survey	8.43 × 10^−155^
Random Forest	Social media	4.47 × 10^−18^
Random Forest	Survey	2.05 × 10^−88^
XGBoost	Social media	3.57 × 10^−59^
XGBoost	Survey	5.49 × 10^−99^
**Unsure**		
Linear	Social media	1
Linear	Survey	9.99 × 10^−1^
Random Forest	Social media	9.99 × 10^−1^
Random Forest	Survey	9.99 × 10^−1^
XGBoost	Social media	9.99 × 10^−1^
XGBoost	Survey	3.78 × 10^−1^
**Hesitant or unsure**		
Linear	Social media	3.07 × 10^−24^
Linear	Survey	1.79 × 10^−144^
Random Forest	Social media	1.56 × 10^−4^
Random Forest	Survey	1.42 × 10^−50^
XGBoost	Social media	1.90 × 10^−25^
XGBoost	Survey	1.00 × 10^−107^
**Probably not**		
Linear	Social media	2.33 × 10^−78^
Linear	Survey	3.26 × 10^−128^
Random Forest	Social media	1.47 × 10^−43^
Random Forest	Survey	2.98 × 10^−52^
XGBoost	Social media	3.42 × 10^−58^
XGBoost	Survey	2.79 × 10^−58^
**Strongly hesitant**		
Linear	Social media	1.72 × 10^−85^
Linear	Survey	5.43 × 10^−139^
Random Forest	Social media	2.68 × 10^−13^
Random Forest	Survey	8.84 × 10^−62^
XGBoost	Social media	9.05 × 10^−28^
XGBoost	Survey	4.06 × 10^−80^

### Model generalizability to metropolitan statistical areas

Table 5 shows the performance, measured by *R*^2^, of the models applied to their respective MSAs. The table highlights the performance variation across each MSA. Negative *R*^2^ values in this context indicate that the model’s performance is below average, reflecting poor performance. For Miami, Los Angeles, and Phoenix, the performance of all models are particularly poor in this respect. These MSAs had 4, 2, and 2 counties respectively compared to other the number of counties in other MSAs that were in the range of 12 to 54. In this case, the lower amount of counties participating in the training data may skewed the performance to MSAs with a larger number of counties. For the remaining MSAs, models’ performance is much higher, with values exceeding 82% on average for random forest and XGBoost. This trend aligns with the patterns discussed in the previous section, where XGBoost consistently outperformed linear regression and random forest methods. It is important to highlight that linear regression is consistently the least effective modeling method in terms of performance.

**Table 5:** MSA model performance (Bolded adjusted *R*^2^ values represent the best performing model for each modelling technique and MSA).

Method	Model	Atl	Chi	Dal	DC	Hou	LA	Mia	NYC	Phl	Phx
Hesitant											
Linear	Baseline	-0.331	0.283	0.211	-1.973	0.495	-27.81	-1.855	0.449	0.273	**0.941**
Linear	Social media	0.517	**0.658**	0.530	0.076	0.650	**0.591**	-45.77	0.699	**0.480**	-0.259
Linear	Survey	**0.613**	0.633	**0.631**	**0.309**	**0.755**	-8.162	-23.16	**0.782**	0.376	0.178
Random Forest	Baseline	**0.889**	0.851	0.840	0.535	0.709	-19.49	-1.499	0.945	0.787	**0.537**
Random Forest	Social media	0.877	0.885	**0.940**	0.723	0.839	-0.223	**0.684**	**0.961**	**0.963**	-4.634
Random Forest	Survey	0.806	**0.971**	0.927	**0.932**	**0.924**	**0.621**	-5.010	0.958	0.954	-1.668
XGBoost	Baseline	**0.918**	0.952	0.763	**0.962**	0.659	0.931	**0.988**	0.970	0.985	**0.999**
XGBoost	Social media	0.914	**0.995**	0.934	0.899	**0.901**	0.974	-1.738	0.957	**0.992**	-3.026
XGBoost	Survey	0.871	0.921	**0.979**	0.849	0.762	**0.996**	0.570	**0.972**	0.987	**0.999**
Unsure											
Linear	Baseline	0.515	0.342	0.108	0.554	0.237	-11.75	-0.298	0.512	**0.451**	-25.29
Linear	Social media	0.468	0.424	0.062	**0.581**	0.301	**0.536**	-1.613	**0.526**	0.433	-7.787
Linear	Survey	**0.644**	**0.535**	**0.222**	0.479	**0.422**	-8.372	**0.348**	0.464	0.450	-14.36
Random Forest	Baseline	0.808	0.732	0.795	0.939	**0.941**	-2.660	0.953	0.896	0.846	-1.314
Random Forest	Social media	0.787	0.835	**0.954**	**0.949**	0.863	-2.022	0.952	**0.948**	**0.935**	-6.930
Random Forest	Survey	**0.848**	**0.896**	0.949	0.846	0.719	-16.27	**0.976**	0.904	0.929	-6.406
XGBoost	Baseline	**0.875**	0.941	0.782	0.889	0.955	-4.445	0.981	0.906	0.961	0.988
XGBoost	Social media	0.812	0.447	**0.991**	**0.959**	**0.993**	**0.940**	**1.000**	0.911	0.961	-1.687
XGBoost	Survey	0.809	**0.970**	0.976	**0.959**	0.807	0.885	0.989	**0.980**	**0.982**	**0.997**
Hesitant or Unsure											
Linear	Baseline	-0.054	0.250	-0.226	-0.005	0.541	-12.38	-2.045	0.413	-0.128	-6.389
Linear	Social media	0.542	0.569	0.343	0.437	**0.895**	**0.699**	-16.80	0.261	**0.007**	-28.28
Linear	Survey	**0.700**	**0.654**	**0.465**	**0.604**	0.773	-3.452	-1.801	**0.608**	-0.557	-0.669
Random Forest	Baseline	0.839	**0.936**	0.910	0.672	**0.953**	-4.641	-0.980	**0.900**	0.768	-1.139
Random Forest	Social media	0.854	0.904	0.871	0.875	0.935	-4.434	**0.821**	0.858	**0.836**	-0.436
Random Forest	Survey	**0.909**	0.848	**0.911**	**0.889**	0.939	**0.358**	0.494	0.868	0.695	**0.269**
XGBoost	Baseline	0.932	0.970	**0.970**	0.964	0.831	**0.969**	0.987	0.958	**0.920**	0.907
XGBoost	Social media	0.841	**0.999**	0.913	0.963	0.713	0.152	**0.995**	0.899	0.819	-14.84
XGBoost	Survey	**0.952**	0.901	0.920	**0.978**	**0.974**	0.022	-0.324	**0.990**	0.905	**0.998**
Probably not											
Linear	Baseline	-1.001	0.253	-0.171	-1.247	0.315	-0.938	-1.808	0.413	0.607	-167.1
Linear	Social media	0.305	0.327	0.065	**0.275**	0.452	-7.702	-79.68	0.492	**0.710**	-30.51
Linear	Survey	**0.373**	**0.727**	**0.388**	0.252	**0.843**	-13.82	-19.21	**0.741**	0.339	-136.2
Random Forest	Baseline	0.756	0.856	0.879	0.650	0.882	**0.770**	-0.479	0.972	0.927	-1.385
Random Forest	Social media	**0.895**	0.895	0.871	0.875	**0.935**	0.437	**0.731**	**0.979**	0.877	**0.156**
Random Forest	Survey	0.807	**0.970**	**0.885**	**0.959**	0.878	-1.575	0.590	0.980	**0.986**	-6.740
XGBoost	Baseline	0.689	0.941	**0.978**	0.609	0.955	**0.914**	**0.915**	**0.982**	0.928	**0.259**
XGBoost	Social media	0.922	0.447	0.767	**0.977**	**0.993**	0.754	0.732	0.968	**0.981**	-4.122
XGBoost	Survey	**0.952**	**0.970**	0.976	0.931	0.807	0.781	-0.492	0.973	0.922	-3.368
Strongly Hesitant											
Linear	Baseline	-0.134	0.511	0.042	-1.457	0.529	-398.6	-2.061	0.726	0.382	-3.219
Linear	Social media	0.572	0.833	0.501	0.030	**0.676**	-12.52	-30.01	0.710	**0.324**	-9.771
Linear	Survey	**0.578**	**0.907**	**0.532**	**0.088**	0.654	-116.7	-12.20	**0.802**	0.215	-2.399
Random Forest	Baseline	0.809	0.908	0.794	0.322	0.943	-174.3	-11.65	0.934	0.783	-2.019
Random Forest	Social media	**0.845**	**0.969**	0.594	0.880	**0.906**	-23.54	**0.478**	**0.942**	**0.904**	-0.305
Random Forest	Survey	0.531	0.822	**0.896**	**0.937**	0.866	-11.39	-0.404	0.939	0.697	**0.367**
XGBoost	Baseline	**0.875**	0.907	0.975	0.851	0.659	-324.4	**0.972**	0.842	0.992	0.987
XGBoost	Social media	0.812	0.736	**0.994**	-0.325	**0.901**	0.471	-0.233	0.707	**0.993**	**0.999**
XGBoost	Survey	0.809	**0.996**	0.867	**0.917**	0.762	**0.811**	0.642	**0.872**	0.901	-4.983

Notably, while the distinction between the performances of XGBoost and random forest is evident, the margin of differentiation is comparatively small. Furthermore, in line with earlier findings, models incorporating survey data consistently demonstrate superior performance when juxtaposed against baseline models or models relying on social media data.

### Determinants of vaccine hesitancy

Vaccine hesitancy poses a complex and multifaceted challenge that is influenced by multiple contributing factors. Those factors pertaining to the models investigated within this study are outlined in Table 6. The table highlights the diverse array of factors operating within distinct models. In a descending order based on the number of variables are the hesitant or unsure (16 variables), unsure (14 variables), hesitant (13 variables), probably not (7 variables), and strongly hesitant (7 variables) models respectfully. The factors associated with the hesitant model primarily revolve around levels of education, cohabitation, occupation, income, political inclinations, as well as the prevalence of COVID-19 cases and vaccination rates. A similar pattern is observed in the unsure model, which also includes ethnicity. On the other hand, the hesitant or unsure model encompasses a broader spectrum of factors, utilizing most variables examined in this study, and span categories that include age, ethnicity, education, cohabitation, occupation, income, political leaning, vaccine distribution, and social vulnerability. Further, the probably not and strongly hesitant models are very similar, sharing factors linked to education, occupation, and income. The probably not model also includes employment status as an important factor.

**Table 6:** Factors related to vaccine hesitancy.

Variable group	Variable	Hesitant	Unsure	Hesitant or unsure	Probably not	Strongly hesitant
Age	Age less than 18 years			✓		
Ethnicity	Black population		✓			
Ethnicity	Asian population			✓		
Education	High school education			✓		
Education	Some college degree	✓		✓	✓	✓
Education	Associate degree					✓
Education	Bachelor degree		✓			
Cohabitation	Couple cohabitation			✓		
Occupation	Manufacturing	✓		✓	✓	✓
Occupation	Professional, scientific, and management, and administrative and waste management service	✓	✓	✓	✓	✓
Occupation	Wholesale trade	✓		✓	✓	✓
Occupation	Sales and office	✓				✓
Occupation	Self employed		✓			
Occupation	Finance and insurance, and real estate and rental and leasing		✓			
Occupation	Transportation and warehousing, and utilities			✓		
Occupation	Private wage and salary workers				✓	✓
Employment	People employed				✓	
Income	Per capita income	✓	✓	✓	✓	✓
Income	Income between 25k and 50k	✓		✓		
Political inclination	Democrat	✓	✓	✓		
Political inclination	Green					
Political inclination	Libertarian	✓	✓	✓		
Political inclination	Other political party	✓	✓	✓		
COVID-19 cases	COVID-19 cases	✓				
COVID-19 vaccination	Completed primary COVID-19 vaccination series	✓	✓			
COVID-19 vaccination	Completed vaccine series against COVID-19		✓	✓		
COVID-19 vaccination	Age 65 years and over that have been administered the first vaccine dose		✓			
Social vulnerability	Social Vulnerability Index		✓			
Social vulnerability	Level of concern	✓	✓	✓		

## Discussion

Vaccine hesitancy is a worldwide phenomena that poses a significant challenge to public health efforts to control or eradicate preventable, but potentially harmful diseases [135]. While not a new issue, the problem has become more endemic in wake of the COVID-19 pandemic, leading to millions of people not being vaccinated globally. [136] estimates that within the first year of the pandemic alone, almost 20 million deaths were averted due to vaccines. Similarly, within the US, between 2020 and 2021, vaccines were estimated to prevent approximately 27 million infections, 1.6 million hospitalizations, and 235,000 deaths [137], with more than 300,000 deaths being prevented the following year [138]. Further, [139] estimates that for every one percent decrease in vaccine hesitancy, as much as 45 deaths per million people could have been prevented during the pandemic, making understanding vaccine hesitancy of key importance for human survival.

One key issue with understanding vaccine hesitancy is with the speed at which data can be gathered and analyzed to provide key insights as events unfold. Traditionally, surveys have been used for this purpose. However, as as discussed earlier on in this paper, this data comes with it’s own set of caveats. In this study we assessed the use of social media data as a potential source of insights on vaccine hesitancy, helping to improve the performance of models used for understanding the determinants surrounding the reluctance to vaccinate when compared to the use of survey data. However, it is important to note that there are also various limitations with the use of social media data on it’s own to understand this issue. For example, certain demographics or socioeconomic groups may be overrepresented or underrepresented [140]. Related is the issue of access bias; not all segments of the population have equal access to social media platforms or may not actively engage in online discussions. This can result in underrepresentation of certain demographics, such as older adults or individuals from low-income communities, in social media data [141]. Social media algorithms may also prioritize content that aligns with some users existing beliefs and preferences, reinforcing pre-existing biases and limiting exposure to diverse perspectives [35]. Moreover, social media data analysis is often conducted in specific languages, which may introduce language bias. Insights drawn from social media data may not be applicable to populations that primarily communicate in different languages, limiting the generalizability of findings [142]. Nevertheless, although various data sources and methodologies may present slightly different viewpoints, together they contribute to a thorough comprehension of the extent and reasons behind vaccine hesitancy at a population level. These varied perspectives function as integral components of a broader puzzle, facilitating the synthesis of insights necessary for developing effective strategies to tackle vaccine hesitancy.

Our results demonstrate that the addition of survey data consistently provides improved model performance compared to social media data across various forms of hesitancy (i.e., hesitant, unsure, probably not, strongly hesitant) and approaches (i.e., linear regression, random forest, XGBoost). However, it’s noteworthy that in some cases, this improvement was marginal, particularly when the XGBoost and random forest techniques were used. This is evidenced by our significance analysis, which demonstrate the robustness of these models in capturing the complexities of hesitancy dynamics.

Additionally, while the generalizability of models to metropolitan statistical areas (MSAs) was generally satisfactory, there were instances of poor MSA performance, with variations observed across different methods. XGBoost still continued to be a robust performer relative to other methods, especially when used in conjunction with survey data. This reinforces the importance of survey data in improving model accuracy beyond the limitations of baseline or social media-derived models. Further, within the same locales and for the top performing models, similar to before, there was marginal difference in model performance between models that use survey data and those that use social media data, highlighting the promise of social media data as a valid source data for understanding vaccine hesitancy. Moreover, the observed performance variations across different MSA locales indicate the influence of additional factors and unique attributes associated with each locale on model outcomes.

Further, congruent with other work, we found age, ethnicity, education, cohabilitation, occupation, employment, income, political leaning, number of disease cases, and the distribution and level of concern for vaccine distribution to be key factors in understanding vaccine hesitancy. It’s important to note, however, that these factors were operationalized differently across the various hesitancy models in this study. This underscores the necessity to consider these nuances when interpreting and applying the results to address vaccine hesitancy effectively. Age, in particular, was only applicable to the hesitant or unsure model, and specifically, people less than 18 year of age. Previous work have explored the relationship between age and ethnicity. For instance, a study by [143] in Ireland and the UK found heightened reluctance to vaccine in age groups 35-45 and 18-24 for Ireland and UK respectfully. [144] investigated schoolchildren aged 9-18 and found considerable indecision (37%) about vaccination, while 12.9% answered that they would opt-out to getting a COVID-19 vaccine. The main reasons given for their reluctance to become vaccinated included distrust of vaccines, government agencies promoting their uptake, apprehensions about side effects, and perceptions of low personal risk. Notably, those opting against vaccination demonstrated higher degrees of marginalization and skepticism towards vaccine information, highlighting the necessity for greater government intervention in addressing these concerns.

The role of ethnicity, specifically the representation of black and Asian populations (primarily originating from East and Southeast Asia, as well as the Indian subcontinent), emerged as a significant determinant within the unsure and hesitant or unsure models. Previous research has extensively explored ethnicity, particularly within the context of BAME (black, Asian, and minority ethnic) communities [145–147]. As pointed out by [148], hesitancy within this group is in part attributed to factors stemming from their exclusion in clinical vaccine trials [148]. Within this context, however, few studies have examined Asians as an independent subgroup within the broader framework of BAME research. [149], who focused on the Asian population as a distinct group in the US, found lower hesitancy compared to Black and Hispanic groups. Similarly, a national survey on COVID-19 vaccine intent among US racial and ethnic groups by [150] revealed that Asian Americans exhibited the lowest refusal rate (11%), in contrast to Black African Americans (32%) and American Indian/Alaska Native respondents (29%). Further research by [151], concentrating on ethnic minorities in a longitudinal study of UK households, found notably varying levels of vaccine hesitancy within Black and Pakistani/Bangladeshi ethnic groups. Exploring the context of the Black population, [152] indicated that higher rates of vaccine hesitancy among Black African Americans. According to [146], this is in part attributed to a lack of trust in medical institutions and concerns about racial injustice.

Turning now towards education, our findings show a general agreement between educational attainment and hesitancy across models. Specifically, they show how even a high school education can influence vaccine indecision, while some college education emerges as the most notable factor in most models. These findings support other related work in this area. For instance, [153] demonstrated heightened hesitancy within less-educated communities in high-income countries. Similarly, [112] observed that individuals with a high school level of education or lower were more inclined to exhibit vaccine hesitancy. Other work by [154], in a survey involving parents in Utah, US, noted that a significant portion of hesitant parents were from the middle-class and possessed either some college education or a college degree. Furthermore, several studies have corroborated that individuals with higher education levels or greater affluence tend to exhibit higher levels of vaccine hesitancy or even a refusal to be vaccinated altogether (e.g., [155–157]).

As it relates to individual household determinants, those with cohabitating couples also emerged as a significant factor to the hesitant or unsure, and strongly hesitant models respectively. Despite the limited amount of research in this area, most of the existing work show a decrease in hesitance rates with cohabilitation (e.g., [158–161]. One exception to this is the work of [162], which in addition to demonstrating increased vaccine hesitancy with cohabilitation, also found being a member of the black population and having less than a college education to be important factors. Additionally, research conducted by [163], focusing on pregnant women in California, indicated that women with partners classified as essential workers exhibit a higher likelihood of hesitancy. The authors of this study propose that essential workers may have been previously exposed to the disease, leading respondents to believe they may have already developed immunity against COVID-19.

Another noteworthy factor gathered from our findings was occupation. The majority of variables within this category are associated with roles that support the day-to-day operations of various businesses, based on occupational classification data provided by the US Bureau of Labor Statistics [91]. Across most models, these trends manifest within three overarching occupational groups: Manufacturing, Professional, scientific, and management, and administrative and waste management services, and Wholesale trade. Work on occupation by [164] have reported more hesitant populations among older workers (aged 40 to 59) in sectors such as service and manufacturing, along with those who are unemployed. Similarly, [165], in a national survey encompassing Japanese adults aged 20 and older, observed reduced likelihood of vaccination among scientists and researchers. Temporal changes in hesitancy across different professions have also been explored in research. For instance, [166] examined shifts in hesitancy rates among various professions between January to May, and April to May 2021. The study highlighted substantial increases in categories such as computer/mathematical (7.3%), educators (9.0%), healthcare practitioners/technicians, and construction/extraction (45.2%) professionals. Furthermore, investigations have underscored variations in hesitancy levels within different healthcare groups [167]. It’s important to note, however, that much of the existing work on vaccine hesitancy and occupation primarily focuses on roles within the medical sector, often classified as essential workers. Nevertheless, there remains limited research in this field [164]. On a related note, being employed was exclusively applicable to the probably not model. These results are corroborated by the findings of other work such as [48, 152]. However, there has been mixed findings in this respect (e.g., [168]) with further research needed to explore this issue.

Furthermore, income was shown to be an important factor, and in particular, per capita income, which displayed significance across all models. Previous investigations into per capita income have reported higher vaccine hesitancy among lower-income groups in comparison to their higher-income counterparts [169, 170]. Additionally, [171], in a survey focusing on parents with children aged 2 to 18, discovered that households with incomes under $100,000 exhibited lower vaccination likelihood than those with incomes of at least $150,000. In a related work, [172] identified families with both low education and income as reporting reduced willingness to vaccinate their children. Moreover, [173] established that the odds of vaccine hesitancy were twice as likely among individuals with middle income compared to those with lower income. Further, [174] analyzing households pre- and post-pandemic found that those who had an income of $100,000 or more prior to the pandemic and experienced income loss during the pandemic displayed heightened levels of hesitancy compared to those who didn’t face income loss. As for the income range of $25,000 to $50,000 annually, these were only applicable to the hesitant and hesitant or unsure models, respectively.

Political leaning was a factor applicable to only three models: hesitant, unsure, and hesitant or unsure, affecting the the same groups. These inclinations encompassed affiliations with the Democratic, Green, Libertarian, and other political parties, which tended to be of lesser prevalence. Research into vaccine hesitancy has delved into these political associations. The majority of studies indicate that Democrats exhibit lower levels of vaccine hesitancy compared to their Republican counterparts. For instance, a study by [175] conducted on Americans revealed that 90% of surveyed Democrats had been vaccinated, while 68% of Independents and 58% of Republicans displayed more hesitancy. Correspondingly, [135] found that Republican members were more inclined to oppose COVID-19 vaccination compared to Democrats. Additional research by [176] suggests that individuals with conservative views are less likely to trust scientific and medical experts, demonstrating a greater inclination to perceive vaccines as unsafe [177] and as a significant health threat [178]. These findings align with the conclusions drawn by [179], which identified that Conservatives and Republicans exhibited higher hesitancy levels compared to their Libertarian and Democrat counterparts.

The final set of variables concerns health, the level of preparedness, and the degree of concern regarding vaccine distribution at various locations. Most of these variables specifically apply to the uncertain model, while all models acknowledge the significance of the level of concern. Previous research has reported an association between vaccine uptake in hesitant communities and their level of apprehension regarding the accessibility and distribution of vaccines [111, 112]. To this end, regions facing greater hurdles in distributing vaccines, as gauged by the multidimensional CVAC index of concern, tend to exhibit lower vaccination rates compared to regions with fewer obstacles [180]. In terms of variables related to the number of COVID-19 cases and primary vaccinations, these findings are relevant to the hesitant and unsure models. Furthermore, the unsure model identifies the completion of a vaccination series as a notable factor. Conversely, social vulnerability is only pertinent to the uncertain model.

The above findings emphasize the complex interplay of diverse variables in shaping vaccine hesitancy. Factors such as education, ethnicity, age, and political orientation were crucial determinants across multiple models. These results underscore the importance of comprehensively understanding these factors to develop effective strategies for addressing vaccine hesitancy and promoting widespread vaccination. However, while this study focused on COVID-19 vaccines, the findings might not be generalizable to other vaccines for a variety of reasons. These concerns may stem from various factors such as their novelty, the short timeline for clinical trials, and the utilization of new mRNA technology in some of them.

With this being said, our work provides several valuable contributions. First, while the addition of the survey data resulted in greater model performance in comparison to the social media data, the speed and scale at which social media data can be collected and analysed makes it a supplementary source of data/insights on vaccine hesitancy. Second, and related, the results highlight limitations in using social-based data alone in understanding vaccine hesitancy. In this case, with the addition of the social media or survey data to the social-base data only models; this resulted in improved performances, as much as 17%. Third, there is an important link with respect to what is discussed online in cyber-social communities and the characteristics of the people in the real world. Finally, our approach lays the groundwork for other similar studies that seek to understand and compare the use of social media and survey data for other topics such as climate change and the economy.

There were also several limitations identified in this work that provide areas of future work. First, only 10 MSAs were examined in this study. Future work should therefore examine additional areas and at greater levels of spatial granularity. Second, working with text data is challenging, with the potential for different people to interpret the hesitance within text differently. In this study we did not assess the quality/agreement of the labelled data, which could be a source of bias in the results. Third, additional sources of social media [181] and survey data should be investigated, along with different machine learning algorithms [61, 72]). Fourth, as has been investigated in other research, other metrics derived from social media, such as sentiment (e.g., [61]) and stance (e.g., [51]), could be incorporated in a similar analysis. Fifth, this study exclusively used English tweets; a similar study encompassing multiple languages would therefore be interesting. Sixth, extending this study to encompass different countries would yield valuable insights, considering the potential variations in hesitancy dynamics across diverse cultural and societal contexts.

Finally, it’s important to note that individuals’ decision to vaccinate or not is influenced by various factors, including individual capabilities, external opportunities, motivations, beliefs about necessity and concerns, and perceptions of health threats. Future work should therefore explore these results in the context of related frameworks, such as the Capability, Opportunity, Motivation, and Behavior Framework [182], the Necessity-Concerns Framework [183, 184], and the Health Belief Model [185], to gain a deeper understanding of the complex factors influencing vaccine hesitancy and inform targeted interventions to address this critical public health issue. Even with these areas for further exploration, this paper demonstrates the utility of utilizing both surveys and social media data in understanding vaccine hesitancy across different locations.
